# Lysosomal Abnormalities in Cardiovascular Disease

**DOI:** 10.3390/ijms21030811

**Published:** 2020-01-27

**Authors:** Congwu Chi, Andrew S. Riching, Kunhua Song

**Affiliations:** 1 Division of Cardiology, Department of Medicine, University of Colorado Anschutz Medical Campus, Aurora, CO 80045, USA; congwu.chi@cuanschutz.edu (C.C.); andrew.riching@cuanschutz.edu (A.S.R.); 2 Gates Center for Regenerative Medicine and Stem Cell Biology, University of Colorado Anschutz Medical Campus, Aurora, CO 80045, USA; 3 The Consortium for Fibrosis Research & Translation, University of Colorado Anschutz Medical Campus, Aurora, CO 80045, USA; 4 Linda Crnic Institute for Down Syndrome, University of Colorado Anschutz Medical Campus, Aurora, CO 80045, USA

**Keywords:** lysosome function, cardiovascular disease, autophagy, Danon disease

## Abstract

The lysosome, a key organelle for cellular clearance, is associated with a wide variety of pathological conditions in humans. Lysosome function and its related pathways are particularly important for maintaining the health of the cardiovascular system. In this review, we highlighted studies that have improved our understanding of the connection between lysosome function and cardiovascular diseases with an emphasis on a recent breakthrough that characterized a unique autophagosome-lysosome fusion mechanism employed by cardiomyocytes through a lysosomal membrane protein LAMP-2B. This finding may impact the development of future therapeutic applications.

## 1. Introduction

The lysosome is a membrane-bound cell organelle that contains digestive enzymes. Lysosomes break down many biomolecules and organelles and, therefore, are involved in a multitude of cellular processes. Thus, lysosome-mediated degradation is considered to be one of the major mechanisms by which cells degrade long-lived or damaged proteins and organelles [1]. Consistent with its key functions in maintaining cellular hemostasis, defects in lysosome functions lead to the development of many human diseases, including cardiovascular diseases [2].

Cardiovascular disease is the leading global cause of death. According to the 2018 report [3], the American Heart Association estimated that cardiovascular disease accounts for more than 800,000, or approximately one in three, deaths in the United States each year. Although there are many causative factors that contribute to the pathogenesis of cardiovascular diseases, lysosome function is tightly correlated with the progression of these diseases [4].

This review focused on the recent advancement of our understanding of the lysosome’s roles in cardiovascular diseases. We then highlighted new mechanisms by which lysosomes contribute to the pathogenesis of cardiovascular diseases and discuss potential therapeutic strategies for clinical applications.

## 2. Lysosome Dysfunction and Cardiovascular Diseases

Lysosomes have long been established as the degradative end points for intracellular cargoes, including unfolded proteins, damaged organelles, and nucleic acids. The degradative function of the lysosome is accomplished by over 60 proteases, lipases, nucleases, and other hydrolytic enzymes that break down cellular cargoes for immediate recycling or storage [5]. Many cellular events, such as endocytosis and autophagy, use lysosomes as the common terminal nexus [6]. Therefore, lysosome dysfunction has detrimental impacts on many cellular processes.

Lysosome dysfunction has been characterized in numerous cardiovascular diseases [4]. Many studies have provided insights into how the lysosome functions in the context of the cardiovascular system. One major category of lysosome dysfunction that contributes to cardiovascular disorders is known as lysosomal storage disorders (LSDs). LSDs comprise a group of diseases caused by a deficiency of lysosomal enzymes, membrane transporters, or other proteins involved in lysosomal biology [7]. Many LSD patients show very severe cardiac phenotypes, including hypertrophic and dilated cardiomyopathy, coronary artery disorders, and valvular defects. The causative factors of these LSDs are various and many mutations in lysosomal genes have been identified as responsible for the disease pathogenesis. For example, Pompe disease is caused by mutations in the acid α-glucosidase (*GAA*) gene that lead to intralysosomal accumulation of glycogen [8]. Danon disease is caused by a deficiency in lysosomal-associated membrane protein-2 (*LAMP2*), a gene which encodes for a lysosomal membrane protein on chromosome X [9,10]. A deficiency in the lysosomal enzyme alpha-galactosidase A (a-Gal A) causes Fabry disease due to buildup of the fatty acid globotriaosylceramide (Gb3) in the body [11]. Mucopolysaccharidosis (MPS) is a large group of storage diseases caused by impaired lysosomal degradation of glycosaminoglycans (GAGs). Multiple mutations in genes encoding lysosomal enzymes involved in the catabolism of GAGs have been associated with MPS. Moreover, heart and valve defects are present in all MPS types and heart failure is a common cause of lethality in MPS [12]. Due to the broad range of lysosomal components that can cause LSDs, the cellular mechanisms underlying pathogenesis of different diseases could be highly disease specific. For example, impairment in autophagy, a type of lysosome-related pathway, plays a causative role in Danon disease [9]. The defective autophagosome-lysosome fusion in Danon cells causes accumulation of damaged mitochondria and, in turn, compromises the energy metabolism of these cells.

In addition to lysosomal components, regulation of lysosome biogenesis is also tightly linked to the development of cardiovascular diseases. The transcription factor EB (TFEB) is a master regulator of lysosome biogenesis [13], which coordinates expression of lysosomal hydrolases, membrane proteins, and genes involved in lysosome biogenesis. Subcellular localization of TFEB is strictly controlled. Under nutrient-rich conditions, TFEB is mainly localized in the cytosol. However, upon starvation or under conditions of lysosomal dysfunction, TFEB rapidly translocates to the nucleus and activates the transcription of its downstream targets [14]. Proteotoxicity and poor protein quality control have been observed in the vast majority of human hearts with end-stage heart failure, which may contribute to disease progression [15,16]. Pan et al. reported that myocardial TFEB expression is dysregulated in mice with advanced cardiac proteinopathy [17]. In this study, the authors found that TFEB is required for sustaining lysosome-associated cellular clearance. Moreover, TFEB overexpression is sufficient to facilitate this activity and thereby protects against misfolded protein-induced proteotoxicity. In myocardial ischemia-reperfusion (I/R) injury, programmed cell death and/or necrosis cause substantial cardiomyocyte (CM) loss. The forced expression of TFEB attenuates CM death through restoration of lysosome-associated cellular clearance [18]. Consistent with this finding, another study using a drug to boost TFEB activity also provided evidence that increased lysosome biogenesis has beneficial effects on the cardiovascular system. In a rat model of myocardial I/R injury, cilostazol (an anti-tumor drug) treatment enhanced the transportation of TFEB to the nucleus and dramatically decreased the infarct size. On the other hand, TFEB inhibition with the compound CCI-779 abolished the protective effects of cilostazol in this I/R injury rat model [19]. In most cases, blocked or reduced lysosome function can cause cardiovascular diseases. However, it has also been observed that, under certain conditions, high lysosomal activity is also deleterious (Table 1). Hyperactivation of lysosome function has been implicated in some cardiovascular complications. The lysosomal cathepsins are a subgroup of cysteine proteases composed of 11 members (cathepsin B, C, F, H, K, L, O, S, V, X, and W). They are mainly localized within the lysosome, contributing to lysosomal degradation of cellular substrates. Under certain pathological conditions, both the expression level and subcellular localization of these cathepsins can change and become detrimental. Cathepsins have been associated with a variety of diseases, including heart failure [20]. Increased cathepsin gene expression was reported in conditions of cardiac stress, remodeling, and dysfunction [21]. Expression of cathepsins S and K was increased in the myocardium of hypertensive rodents and in humans with hypertension-induced heart failure [22]. Elevated cathepsin D was found in the plasma of patients after myocardial infarction [23]. In a clinical trial study, higher circulating serum cathepsin B was associated with an increased risk of a composite outcome of specific cardiovascular events or all-cause mortality in 4372 patients with stable coronary heart disease [24]. In a mouse MPS model, Gonzalez et al. found that cathepsin B plays a role in remodeling the extracellular matrix (ECM) and the pathogenesis of MPS type I. Secreted cathepsin B may consequently degrade the ECM in the heart and cause the cardiac phenotypes observed [25].

In addition to genetic cues, cardiotoxic chemicals, such as doxorubicin, can also induce lysosome dysfunction that in turn contribute to disease progression. Doxorubicin is a very effective and widely used chemotherapeutic drug. The anti-tumor effect of doxorubicin has been primarily attributed to its ability to intercalate into DNA, thereby blocking DNA replication and mRNA transcription [26]. However, its clinical use is limited by dose-dependent cardiotoxicity, which can lead to dilated cardiomyopathy (DCM) and congestive heart failure (CHF) [27]. Many mechanisms are associated with the cardiotoxicity of doxorubicin, including increased oxidative stress, decreased levels of antioxidants and sulfhydryl groups, inhibition of nucleic acid and protein synthesis, release of vasoactive amines, altered adrenergic function, and decreased expression of cardiac-specific genes [27]. A recent study highlighted a new mechanism by which doxorubicin impacts lysosomal function. Using both in vivo and in vitro systems [28]. it was discovered that doxorubicin inhibits lysosome function by alkalinizing lysosomal pH. The activities of most lysosomal enzymes are tightly regulated by pH. The acidic pH of the lysosome is maintained mainly through the activity of the Vacuolar-type H+-ATPase (V-ATPase). Further study identified that doxorubicin treatment suppressed the activity of V-ATPase [28]. This study provided a novel mechanism of doxorubicin cardiotoxicity and the interaction between cardiotoxic chemicals and lysosome function.

## 3. Phenotypes of Danon Cardiomyopathy

Danon disease is a lysosomal storage disorder and manifests in patients as cardiomyopathy, skeletal muscle weakness, and intellectual disability. Previous studies have discovered that Danon disease is associated with mutations in the *LAMP2* gene, which is located on the human X chromosome and encodes a lysosomal membrane protein [29,30]. Due to the X-linked inherited nature of the *LAMP2* gene, male patients with Danon disease usually develop the disease earlier in life (in childhood or adolescence) and display more severe phenotypes than female patients. Many male patients die of progressive heart failure at the average age of 19 without a heart transplant. However, female patients develop the disease later in life and present with less severe phenotypes [30].

Female Danon patients carrying both wildtype (WT) and mutant *LAMP2* alleles (heterozygous) often display divergent clinical phenotypes. Studies using CMs derived from human-induced pluripotent stem cells (hiPSC-CMs) offer a unique platform to further study this phenomenon. Two diverse populations of hiPSCs can be generated from heterozygous female patients with Danon disease: one with WT LAMP-2 expression and the other with mutant LAMP-2 expression, which is due to the random inactivation of the X chromosome carrying the WT or mutant *LAMP2* [31]. These two hiPSC-CM populations showed distinct phenotypes. Only the hiPSC-CM population carrying the mutant *LAMP2* allele on the active X chromosome demonstrated the in vitro phenotypes of Danon disease. DNA methylation was correlated with X chromosome inactivation [32]. Ng and colleagues used 5-aza-2′-deoxycytidine, which inhibits DNA methyltransferase activity, to reactivate the silenced X chromosome bearing the WT *LAMP2* allele. The treatment with 5-aza-2′-deoxycytidine partially restored WT LAMP-2 expression in female Danon hiPSC-CMs, leading to increased contractility and decreased accumulation of autophagosomes [33]. Reactivation of the X chromosome therefore holds therapeutic potential for female patients with Danon disease [33].

A previous report has shown that CM apoptosis could be a potential causative factor that contributes to Danon pathogenesis [34]. Hashem and colleagues reported apoptosis, which was induced by the excessive amount of reactive oxygen species (ROS) produced by the mitochondria, significantly increased in hiPSC-CMs from male Danon patients compared to the control. However, whether CM apoptosis plays a major role in Danon pathogenesis is not clear since no significant increase of hiPSC-CM apoptosis was detected in more recent studies [9,31].

## 4. Defective Autophagy Correlates to Danon Cardiomyopathy

Numerous cardiovascular diseases exhibit defects in autophagy, including Danon disease [29]. Autophagy is a process of self-cannibalization by which cells recycle misfolded proteins and damaged organelles. The resulting breakdown products serve as inputs for energy metabolism and allow cells to produce more energy to deal with starvation or stress. Three main types of autophagy have been characterized thus far: microautophagy, chaperone-mediated autophagy (CMA), and macroautophagy. Microautophagy involves the direct uptake of soluble or particulate cellular cargoes into lysosomes via invagination, protrusion, or septation of the lysosomal membrane. Microautophagy is the least studied form of autophagy [35,36]. The detailed mechanism of microautophagy and its contribution to the cardiovascular system remain elusive. CMA, on the other hand, has been well studied and has been reviewed extensively [37,38]. While the extent of CMA in the cardiovascular system is not fully understood, several proteins with known function in the heart have been identified as CMA substrates, including calcineurin (RCAN1), ryanodine receptor 2 (RYR2) and myocyte enhancer factor 2D (MEF2D) [39,40,41].

In contrast to microautophagy and CMA, macroautophagy has been shown to play key roles in the cardiovascular system, demonstrating both cardioprotective and maladaptive roles in disease [42,43]. Macroautophagy is an intracellular process that relies on the formation of the autophagosome, a double membrane vesicle, which carries cellular cargoes to lysosomes. Autophagosomes then fuse with lysosomes to form autolysosomes in which these cellular cargoes are degraded by lysosome-derived acid hydrolases [44]. This review focused on this form of autophagy, macroautophagy (hereafter referred to as autophagy). Cardiac fibroblasts, CMs, endothelial cells, and vascular smooth muscle cells are the major cellular constituents of the heart [45]. Studies focused on cell types other than CMs have shed light on how autophagy activity contributes to the progression of cardiovascular diseases. For example, in endothelial cells, autophagy involves the regulation of cell survival, nitric oxide production, angiogenesis, and haemostasis/thrombosis [46]. Unlike the other three major cell types in the heart, CMs have very limited regenerative capacity due to their postmitotic state [47]. The house-keeping function provided by autophagy is particularly critical for post-mitotic and terminally differentiated cells, including CMs. These cells must survive for many years and cannot dilute the accumulation of cellular waste by cell division due to low turnover rate. Therefore, dysregulation of autophagy is detrimental to these cells and contributes to the development of many diseases. Due to the scope of this review, we mainly focused on discussing the contribution of autophagy in CMs. 

The cardiovascular system has one of the highest energy demands in the body, consuming as much as 440 kcal per kg per day [48]. Unlike skeletal muscle, heart muscle functions almost exclusively aerobically, as evidenced by the density of mitochondria in CMs. Glycogen, one of the key energy sources in response to cardiac metabolic stress, is degraded via autophagy to be used as a substrate for glucose-mediated ATP production [49,50]. Therefore, autophagy is critical for the heart to maintain the health of its mitochondria and produce enough energy for contractile function.

One of the pathological hallmarks of Danon disease includes the accumulation of intracellular autophagic vacuoles containing glycogen within CMs [51]. Moreover, muscle biopsies from Danon patients and iPSC-CMs derived from Danon patients exhibited defects in autophagic flux [29,31,52]. We recently examined the gene expression profiles of six hiPSC-CM lines derived from three unrelated male Danon patients, two unrelated male healthy controls, and one isogenic LAMP-2 knockout (KO) cell line generated from one of the control lines by CRISPR-Cas9 genome editing technology [9]. Danon hiPSC-CMs recapitulated phenotypes observed in Danon patients and previous studies using Danon hiPSC-CMs, including glycogen accumulation in autophagosome-like vacuoles, suggesting impaired augophagosome-lysosome fusion. Gene ontology analysis demonstrated that 150 out of 420 differentially expressed genes with known functions were involved in metabolic processes, indicating a dramatic metabolic defect in Danon cells. To further understand this metabolic defect, we studied the relationship between defective autophagosome-lysosome fusion we observed in Danon hiPSC-CMs and mitochondrial dysfunction. Mitochondrial dynamics, including fission and fusion, are tightly linked with mitochondrial autophagy (mitophagy) and homeostasis [53]. In Danon hiPSC-CMs, we observed the accumulation of depolarized mitochondria, which in turn decreased global mitochondrial membrane potential. Fission is known to induce mitochondrial depolarization [53]. Consistent with this notion, more mitochondrial fragmentation, which was associated with a high level of the short isoform of optic atrophy 1 (S-OPA1) [54], was detected in Danon hiPSC-CMs than in the controls. These depolarized and fragmented mitochondria become the substrates for mitophagy. We then examined the energy production in Danon hiPSC-CMs. Danon cells produced significantly less cellular ATP than the controls, which is consistent with the notion that mitochondria play a central role in energy metabolism. Taken together, the accumulation of autophagosomes, fragmentation of the mitochondrial network, and reduced ATP production suggest that defects in autophagosome-lysosome fusion strongly inhibit mitophagy in Danon hiPSC-CMs. Interestingly, Danon hiPSC-CMs also displayed decreased contractile twitch force compared to controls measured using a micropost array platform [55]. In conclusion, our study, along with others, shed new light on the pathogenesis of Danon disease by bridging the defect in autophagy with the development of cardiomyopathy.

## 5. Deficiency of the B Isoform of LAMP-2 is the Main Causative Factor of Danon Cardiomyopathy

The *LAMP2* gene produces 3 isoforms, LAMP-2A, LAMP-2B, and LAMP-2C, through alternative splicing. Proteins of these isoforms share an identical N-terminus (lysosome luminal domain) while having distinct C-termini, including transmembrane domains and cytosolic tails. LAMP-2A and LAMP-2B are broadly expressed in many human tissues whereas the expression level of LAMP-2C is low [56]. We examined the expression of LAMP-2 isoforms in several human cell types. LAMP-2B is the predominant isoform, which accounts for over 70% of total LAMP-2 protein in human and mouse CMs. To study the impacts of different LAMP-2 isoforms on Danon pathogenesis, we generated isoform-specific KO lines from an isogenic healthy control line. LAMP-2B KO, but not LAMP-2A or -2C KO hiPSC-CMs recapitulated the phenotypes we observed in Danon hiPSC-CMs, including defects in mitochondria function, energy production, and autophagy. Thus, our study provided strong evidence that the B isoform of LAMP-2 plays a causative role in the development of Danon pathogenesis.

## 6. A Novel Mechanism of LAMP-2B in Autophagosome-Lysosome Fusion in CMs

Extensive studies have established a key role of LAMP-2A in CMA [57,58]. LAMP-2C has been implicated in novel types of autophagy responsible for RNA and DNA uptake and degradation [59,60]. Recently, LAMP-2C has also been shown to function as a negative regulator of CMA in the immune system [56]. Unlike the other two isoforms, LAMP-2B’s role in autophagy remains elusive. Using an autophagic flux assay, we found that only LAMP-2B KO hiPSC-CMs displayed an autophagy defect, which was not observed in LAMP-2A or LAMP-2C KO hiPSC-CMs. Microtubule-associated protein light chain 3 (LC3)-II has been previously shown to mark mature autophagosomes [61]. In our system, LAMP-2B deficiency caused the accumulation of LC3-II-positive autophagosomes, which is due to a dramatically impaired fusion between autophagosomes and lysosomes. Studies using non-CM cells, such as HEK293 cells and mouse embryonic fibroblasts (MEFs), reveal that Syntaxin 17 (STX17) is required for autophagosome-lysosome fusion through its interactions with synaptosome-associated protein 29 (SNAP29) and vesicle-associated membrane protein 8 (VAMP8). Autophagy related 14 (ATG14) enhances autophagosome-lysosome fusion by interacting with the STX17-SNAP29 complex [62,63]. However, knockout of STX17 in hiPSC-CMs did not cause significant accumulation of autophagosomes, suggesting an STX17-independent autophagosome-lysosome fusion mechanism in CMs.

To uncover the molecular role of LAMP-2B in autophagosome-lysosome fusion, we took advantage of 2 cell lines with low (HEK293) or high (hiPSC-CMs) levels of LAMP-2B expression. Ectopic overexpression of LAMP-2B in HEK293 cells significantly enhanced the interaction between ATG14 (on autophagosomes) and VAMP8 (on lysosomes). To our surprise, overexpression of LAMP-2B also suppressed the autophagosome-lysosome fusion defect induced by STX17 knockdown, which indicates that LAMP-2B functions independent of STX17. We further verified these findings in CMs that express high levels of LAMP-2B. ATG14 and VAMP8 formed a complex with endogenous LAMP-2B. This complex was disrupted in Danon or LAMP-2B KO hiPSC-CMs. In conclusion, our findings established a molecular mechanism by which the loss of the lysosomal-membrane protein LAMP-2B disrupts autophagosome-lysosome fusion in Danon disease. Therefore, targeting LAMP-2B represents a potential therapeutic avenue to restore defective autophagy in Danon cardiomyopathy. Moreover, LAMP-2B-deficient hiPSC-CMs could also serve as an in vitro model of Danon disease for drug screening and discovery to identify novel therapeutic agents that restore autophagosome-lysosome fusion.

## 7. Gene Therapy to Treat LSDs

The development of lysosomal storage disorders often occurs from mutations in a single gene. As mentioned, Pompe disease, Danon disease, Fabry disease, and MPS type I are caused by mutations in the *GAA* [8], *LAMP-2* [9,10], α-galactosidase A (a-*Gal A*) [11], and the α-L-iduronidase (*IDUA*) genes [12], respectively. Gene therapy is a promising therapeutic intervention strategy that utilizes viral infection to replace mutated, pathogenic genes with wildtype copies to ameliorate or cure disease. While the concept of gene therapy originated in the 1960s and the first controversial human study was performed in 1980 led by Martin Cline, the safety of gene therapy has been extensively debated since its conception [64,65].

The first clinical trials for gene therapy took place in the 1991 using retroviral delivery to infect patient-derived peripheral blood cells. However, despite X-linked severe combined immunodeficiency being successfully treated by retroviral gene therapy in pediatric patients in 2000, it was later discovered that retroviral insertion into these patients’ genomes directly led to some of these patients developing leukemia [65]. Additionally, delivery of the ornithine carbamoyltransferase gene by adenovirus triggered a severe, ultimately fatal immune reaction in one patient in 1999 [66].

Adeno-associated virus (AAV) has also been used extensively to deliver gene cargo to patients and exhibits low immune response and poor genomic integration into the host genome, suggesting better safety and tolerability in patients compared to retroviral and adenoviral delivery systems [67]. There are 13 serotypes of AAV, which each exhibit preferential binding to cell surface receptors and glycans, thereby altering tissue tropism and allowing for somewhat selective infection of certain organs [68]. Currently, AAV gene therapy safety and efficacy is being tested in over 150 clinical trials for various diseases, including Pompe disease (NCT02240407), Fabry disease (NCT04046224, NCT04040049), MPS types I-III (NCT02702115, NCT03566043, NCT04088734, NCT03612869), and Danon disease (NCT03882437).

While the Food and Drug Administration (FDA) has already approved several AAV therapies [69], safety concerns of gene therapy still exist, especially for the treatment of chronic diseases like lysosomal storage disorders. For example, Danon cardiomyopathy represents a very severe form of cardiomyopathy that has been shown to result from impaired autophagy. Currently, there is no cure for this disease. A new clinical trial (NCT03882437) was recently started to assess the safety and toxicity of LAMP-2B gene therapy delivered by adeno-associated virus serotype 9 (AAV9) in male patients with Danon disease. These patients may need continuous injections over their lifetimes due to the limited duration of AAV-driven overexpression. Furthermore, the degree of transgene overexpression must be finely controlled to avoid toxicity. Moreover, AAV infection has been shown to cause hepatic toxicity and hepatocellular carcinoma in mice [70,71]. While tumor incidence has not been associated with AAV treatment in humans thus far (with >7 year follow up from certain trials) [72], the long-term safety of AAV therapies is still largely unknown. Despite these concerns, gene therapy remains a promising strategy to treat genetic diseases and will undoubtedly continue to improve in the coming decades.

## 8. Perspectives

LSD is an important category of diseases caused by lysosome dysfunction. Current therapeutic strategies targeting LSDs have mainly focused on restoring the activity of defective lysosome function thus far. These strategies include haematopoietic stem cell transplantation, enzyme replacement therapy, substrate reduction, and chaperone therapies [73]. However, these strategies have limitations, including the difficulty of targeting the therapies to the required sites in the body. Furthermore, treatment is usually only initiated after organ damage has already occurred. Therefore, the effectiveness of these therapies still requires further evaluation.

Autophagy, one of the key cellular clearance mechanisms tightly linked to normal lysosome function, plays a critical role in the cardiovascular system. Cardiovascular disorders have been shown to be both positively and negatively influenced by autophagy. Therefore, autophagy represents a promising therapeutic avenue to treat cardiovascular diseases. However, further investigation is required to delineate the specific contexts in which autophagy is salutary or deleterious. Establishing detailed molecular mechanisms into how autophagy affects cardiovascular disease progression is crucial in developing novel therapeutics.

Mechanistic insights into Danon pathogenesis gained from using isoform-specific KO cell lines informed us that the hiPSC-based disease-modeling system holds immense value for future translational studies. By using CRISPR/Cas9 genome editing technology, we generated an isogenic, mutation-corrected hiPSC line from one of the hiPSC lines derived from Danon patients. CMs derived from the mutation-corrected hiPSC line exhibited restored mitochondrial function and autophagic flux as well as significantly improved contractile function compared to the parental Danon line. These data provide a valuable foundation for gene correction-based therapy in patients with Danon disease. To date, 68 different LAMP-2 mutations have been observed in Danon patients [30]. Out of the 68, 64 are point or small insertion/deletion mutations that could potentially serve as targets for CRISPR/Cas9 mediated gene correction.

In addition to CMs, LAMP-2B expression was also highly enriched in the brain, skeletal muscle, and retinal pigment epithelium [74,75]. Consistent with this notion, Danon patients also exhibited mental retardation, skeletal muscle weakness, and vision impairment. Given the high energy consumption of these three organs, a similar LAMP-2B-mediated mechanism of autophagosome/lysosome fusion may exist in these tissues in addition to the heart. Therefore, studies using cells generated from LAMP-2B KO hiPSCs could provide novel insights into the pathogenesis of Danon-derived mental disorder, muscle weakness, and visual defects.

## 9. Conclusions

In this review, we summarized our current view of the relationship between lysosome function and cardiovascular diseases. Evidence provided by recent studies has greatly increased our understanding of how dysregulation of lysosomal pathways in the cardiovascular system contribute to disease in a highly context-dependent manner. Using Danon disease, a disease caused by impaired autophagosome/lysosome fusion and which displays very severe cardiomyopathy phenotypes, as an example, we further discussed how the new insights into the molecular mechanisms of disease pathogenesis could impact the development of novel therapeutic strategies. Fine tuning the activity of lysosomal pathways will be a great challenge due to the dual role of lysosomes in cardiovascular diseases. However, there is no doubt that lysosomal function significantly contributes to disease pathogenesis and therefore represents an important target of future therapeutics.

## Figures and Tables

**Table 1:** Recent studies on lysosome dysfunction in cardiovascular disease.

Lysosome Function	Lysosomal Component/Pathway	Disease/Models	References
Insufficient	V-ATPase/lysosome acidification	Doxorubicin-induced cardiotoxicity	[28]
	TFEB/biogenesis	cardiac proteinopathy/desmin-related cardiomyopathy	[17]
	LAMP-2B/autophagy	Danon disease	[9]
	TFEB/biogenesis	Myocardial I/R injury	[19]
Excessive	Cathepsin B	Coronary heart disease (CLARICOR trial)	[24]
	Cathepsin B	Mucopolysaccharidosis type I	[25]

## Abbreviations

LSDs	Lysosomal storage disorders
GAA	Acid α-glucosidase
LAMP-2	Lysosomal associated membrane protein-2
a-Gal A	Alpha-galactosidase A
Gb3	Globotriaosylceramide
MPS	Mucopolysaccharidosis
GAGs	Glycosaminoglycans
TFEB	Transcription factor EB
CM	Cardiomyocyte
I/R	Ischemia-reperfusion
ECM	Extracellular matrix
DCM	Dilated cardiomyopathy
CHF	Congestive heart failure
V-ATPase	Vacuolar-type H+-ATPase
CMA	Chaperone mediated autophagy
RCAN1	Calcineurin
RYR2	Ryanodine receptor 2
MEF2D	Myocyte enhancer factor 2D
WT	Wildtype
hiPSC-CMs	Cardiomyocytes derived from human induced pluripotent stem cells
ROS	Reactive oxygen species
KO	Knockout
Mitophagy	Mitochondrial autophagy
S-OPA1	Short isoform of optic atrophy 1
LC3	Microtubule-associated protein light chain 3
MEFs	Mouse embryonic fibroblasts
STX17	Syntaxin 17
SNAP29	Synaptosome-associated protein 29
VAMP8	Vesicle-associated membrane protein 8
ATG14	Autophagy related 14
IDUA	α-L-iduronidase
AAV9	Adeno-associated virus serotype 9
FDA	Food and Drug Administration
